# Unigems: plasmids and parts to facilitate teaching on assembly, gene expression control and logic in *E. coli*

**DOI:** 10.1099/acmi.0.000596.v3

**Published:** 2023-09-06

**Authors:** Alex Siddall, Abbie Ann Williams, Jason Sanders, Jai A. Denton, Dean Madden, John Schollar, Jarosław Bryk

**Affiliations:** ^1^​ School of Applied Sciences, University of Huddersfield, Huddersfield, UK; ^2^​ School of Biological Sciences, University of East Anglia, Norwich, UK; ^3^​ Genomics and Regulatory Systems Unit, Okinawa Institute of Science and Technology Graduate University, Okinawa, Japan; ^4^​ School of Art, Design and Architecture, University of Huddersfield, Huddersfield, UK; ^5^​ Institute of Vector-borne Disease, Monash University, Clayton, Australia; ^6^​ National Centre for Biotechnology Education, University of Reading, Reading, UK

**Keywords:** synthetic biology, Gibson assembly, iGEM, plasmids, education

## Abstract

Synthetic biology enables the creative combination of engineering and molecular biology for exploration of fundamental aspects of biological phenomena. However, there are limited resources available for such applications in the educational context, where straightforward setup, easily measurable phenotypes and extensibility are of particular importance. We developed unigems, a set of ten plasmids that enable classroom-based investigation of gene-expression control and biological logic gates to facilitate teaching synthetic biology and genetic engineering. It is built on a high-copy plasmid backbone and is easily extensible thanks to a common primer set that facilitates Gibson assembly of PCR-generated or synthesized DNA parts into the target vector. It includes two reporter genes with either two constitutive (high- or low-level) or two inducible (lactose- or arabinose-) promoters, as well as a single-plasmid implementation of an AND logic gate. The set can readily be employed in undergraduate teaching settings, during outreach events and for training of iGEM teams. All plasmids have been deposited in Addgene.

## Data Summary

The plasmids have been deposited at Addgene (https://www.addgene.org/Jaroslaw_Bryk/). An accompanying Figshare repository (https://figshare.com/projects/Unigems_paper/114069) contains SnapGene sequences of all constructs as well as EPS and Adobe Illustrator files with the plasmid maps to allow educators create their own high-quality figures of their assemblies.

**Impact Statement**

There is a dearth of practical synthetic biology resources available – plasmids, DNA parts and ways to generate and manipulate them – that could easily be implemented in teaching, outreach events or as foundational constructs for more complex designs. We designed a set of ten plasmids that can be broken down to five functional sections (reporter gene and promoter, origin of replication, antibiotic resistance and repressor) using the same set of overlapping PCR primers, therefore making each section compatible with any plasmid in the set. Each of the sections can also be replaced with synthetic DNA parts and/or via PCR with Gibson assembly. We have been using the system in introductory undergraduate synthetic biology laboratory classes, in student research projects, in iGEM teams training and in outreach events and expect they will become a valuable tool for teachers, university instructors and educators. All plasmids and corresponding source files and illustrations are available through Addgene and Figshare.

## Introduction

The development and standardization of biological parts (DNA or RNA fragments) to reliably achieve a predictable output in living organisms has been an often-emphasized aspect of synthetic biology [1]. Remarkable progress has been achieved in characterization of bacterial promoters [2], transcriptional terminators [3], insulators [4], translation optimization [5] and promoter inducibility [6], however advances on a higher level of systems’ complexity have not been as forthcoming [7–9] and major synthetic biology applications (e.g. [10–14]) were achieved by a host of sophisticated and custom-developed genetic engineering [15, 16].

Nevertheless, standardized biological parts are particularly well suited to use in an educational context, as they emphasize utility and remove complexities associated with protocol development [17–19]. These advantages are best illustrated by the BioBuilder lessons, which provide detailed protocols that combine lab-based experiments with engineering tasks to demonstrate fundamental principles of biological and electronic circuits [20] (https://biobuilder.org/), and the International Genetically Engineered Machines competition (iGEM), where thousands of students worldwide create synthetic biology designs and constructs based on and inspired by a set of DNA parts provided by the iGEM Foundation [21]. Notably, practical synthetic biology exemplified by building novel biological constructs from compatible genetic parts, favour active learning approaches in teaching, which were shown to improve students’ academic attainment [22, 23].

To expand the availability of education-friendly resources for synthetic biology we developed unigems, a set of plasmids with simple features such as strong and weak constitutive promoters or single- and dual-inducible promoters and an olfactory construct that can be used directly in laboratory classes. In addition, each plasmid can be split into functional parts that can be exchanged either between the plasmids or replaced with novel parts with PCR and Gibson assembly.

## Methods

### Plasmids

All the plasmids in the unigems set have been assembled using Gibson assembly [24] on the backbone of the pJ401 high-copy plasmid obtained from Atum (Newark, CA, USA). The assembled parts were synthesized by IDT DNA (Coralville, IO, USA) and Life Technologies (now Thermo Fisher Scientific, Waltham, MA, USA).

### Transformation

All transformations were performed using the Sub-cloning Efficiency DH5*α*™ Competent cells (Invitrogen, now Thermo Fisher Scientific, Waltham, USA) as per the manufacturer’s protocol.

### Growth and storage conditions

All cells were cultured using LB Lennox media and agar plates, with incubation at 37^◦^C and agitation at 180 r.p.m. for liquid cultures. Volumes of cultures were 1 or 5 ml, depending on context (smaller for plating transformed cells, sequencing and fluorescence analysis, larger for plasmid isolation). For selective media, both kanamycin and ampicillin (Sigma-Aldrich, now Merck, Darmstadt, Germany) were used at concentration of 50 µg ml^−1^. Plates and cultures were stored at 4^◦^C or preserved for long-term storage at −80^◦^C with 500 µl of overnight culture suspended in 500 µl of 50 % glycerol.

### Gibson assembly

Fragments to be inserted were designed with 20–40 bp overlaps of the vector primer binding sites using SnapGene and synthesized by Integrated DNA Technologies. Platinum SuperFi Green PCR Master Mix (Invitrogen) or Q5 High Fidelity Polymerase (New England Biolabs, Ipswich, MA, USA) were used for generation of backbones from p005 and p006 vectors following the manufacturers’ protocols. PCR products were purified with GeneJET PCR purification kit (Thermo Fisher Scientific) and concentration measured using NanoDrop 2000 Spectrophotometer (Thermo Fisher Scientific). Homemade Gibson Assembly master mix was prepared and assembly carried out as outlined by [25], using 2-3 : 1 of molar ratio of the donor parts to target vectors.

### Plasmid verification

Following transformation, colony PCR was performed using DreamTaq Green PCR Master Mix (2X) (Thermo Fisher Scientific) as described at https://openwetware.org/wiki/Endy:Colony_PCR. Colony PCR products were verified on 1 % agarose gels stained with RedSafe (ChemBio, Oxford, UK) and then purified with GeneJET PCR purification kit (Thermo Fisher Scientific) and sequenced by SourceBioscience (Nottingham, UK).

### Analysis of fluorescence

Horiba FluoroMax−4 spectrofluorometer (Horiba, Kyoto, Japan) was used to obtain emission and excitation spectra for green and red fluorescent proteins. We used 1 ml of overnight cultures resuspended in phosphate buffered saline (PBS, Sigma-Aldrich) to an OD600 below 0.120. Guava easyCyte 5HT system flow cytometer (Merck) was used to determine fluorescence of 10 000 events of each triplicate culture. A signal from RFP cultures, indicating protein expression, was only detected after a 24 h period of incubation.

### Olfactory measurements

The banana smell was identified using the protocol by Dixon and Kuldell [17], following overnight culture at 37^◦^C at 180 r.p.m. to reach stationary growth phase.

## Results

### Assembled plasmids

The unigems' set includes ten plasmids shown in Table 1.

**Table 1.** Unigems plasmids

*Plasmidname*	*AddgeneID*	*Antibiotic resistance*	*Reporter gene*	*Function*
p006-strongGFP	108 313	kanR	GFP	pFAB4026 strong constitutive
p006-weakGFP	108 314	kanR	GFP	pFAB4282 weak constitutive
p005-strongRFP	108 317	kanR	RFP	pFAB4005 strong constitutive
p005-weakRFP	108 316	kanR	RFP	pFAB4024 weak constitutive
p006-pBADGFP	108 315	kanR	GFP	pBAD
p006kanGFP	58 534	kanR	GFP	T5-pLac_O_
p005kanRFP	58 533	kanR	RFP	T5-pLac_O_
p007ampGFP	58 535	ampR	GFP	T5-pLac_O_
p006-ANDGFP	112 237	kanR	GFP	pLac +pBAD AND logic gate
p006-Banana-Late	112 251	kanR	ATFI	osmY

The promoters and terminators are derived from the BIOFAB collection: pFAB4026, pFAB4282, pFAB4005, pFAB4024 [26] and BBa B0062-R [3], respectively. The pBAD-araC and osmY-ATF1 parts come from IGEM repository (BBa K808000, positions 1 : 1200 bp) and BBa J45250, while the AND logic gate is based on the D61 clone from [27]. The RFP reporter gene is a synthetic gene (ID 97752) generated by Atum (Newark, CA, USA) by random assembly, but it has a 76 % sequence identity to an RFP from a strawberry coral *Corynactis californica*. GFP is a standard reporter gene from *Aquorea victoria*. All plasmids also include the lacI repressor under control of a weak constitutive Amp promoter, even when they do not have T5-pLacO promoter themselves.

### The unigems system

**Fig. 1.**
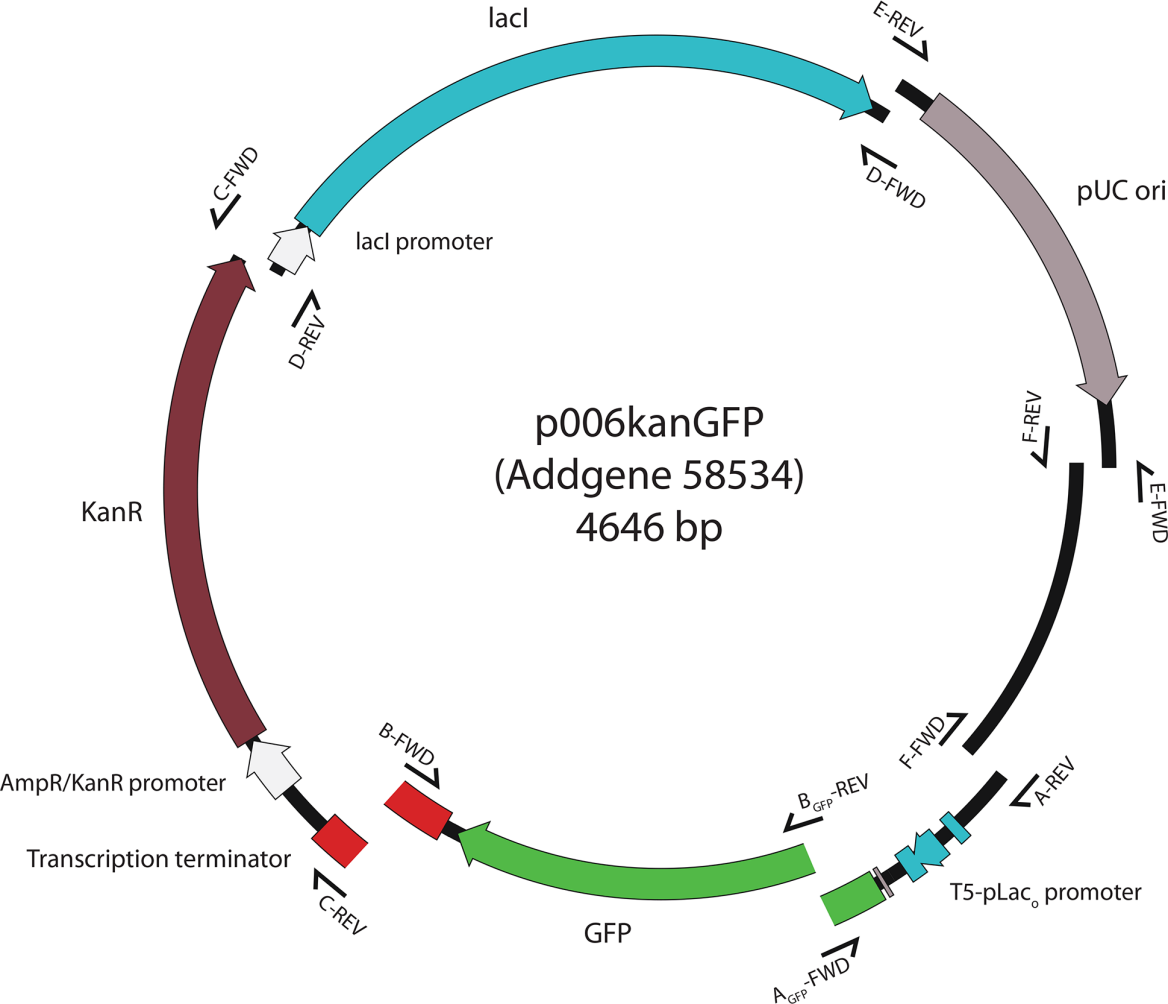
Building sections of the standard unigems plasmid, with the location of each pair of overlapping primer binding sites: primers F-FWD and A-REV, A_GFP_-FWD and B_GFP_-REV, B-FWD and C-REV, C-FWD and d-REV, d-FWD and E-REV, E-FWD and F-REV overlap such that PCR products made with them can be directly used in Gibson assembly. Note that all primer pairs except A_GFP_-FWD and A_GFP_-REV are identical in all plasmids.

The unigems system is built upon a set of six pairs of overlapping PCR primer binding sites (Table 2) that split each plasmid into functional sections that can be replaced or exchanged. Thanks to this arrangement, each plasmid can be a source of parts for other recipient plasmids and new parts can be generated by direct synthesis or PCR with overhanging primers matching the primer binding sites on the unigems plasmids (Fig. 1).

**Table 2.** Primers for the unigems plasmids

Primer name	Primer sequence (5’ → 3’)
A-REV	CTCGAAAATAATAAAGGGAAAATCAG
A_GFP_-FWD	TTCTCCCTCTCCACTGACAG
A_RFP_-FWD	TACGGTTTGCCTGTACCTTC
B-FWD	CTCAGAAGTGAAACGCCGTA
B_GFP_-REV	GGGCACAAATTTTCTGTCAG
B_RFP_-REV	GTACAGGCAAACCGTATGAG
C-FWD	TCACCACCCTGAATTGACTC
C-REV	ACTACCATCGGCGCTACG
d-FWD	CTCACGTTAAGGGATTTTGG
d-REV	CGCCCGGAAGAGAGTC
E-FWD	ACTCAAAGGCGGTAATACGG
E-REV	CCAAAATCCCTTAACGTGAG
F-FWD	CTGATTTTCCCTTTATTATTTTCGAGA
F-REV	CCTGATTCTGTGGATAACCG

**Fig. 2.**
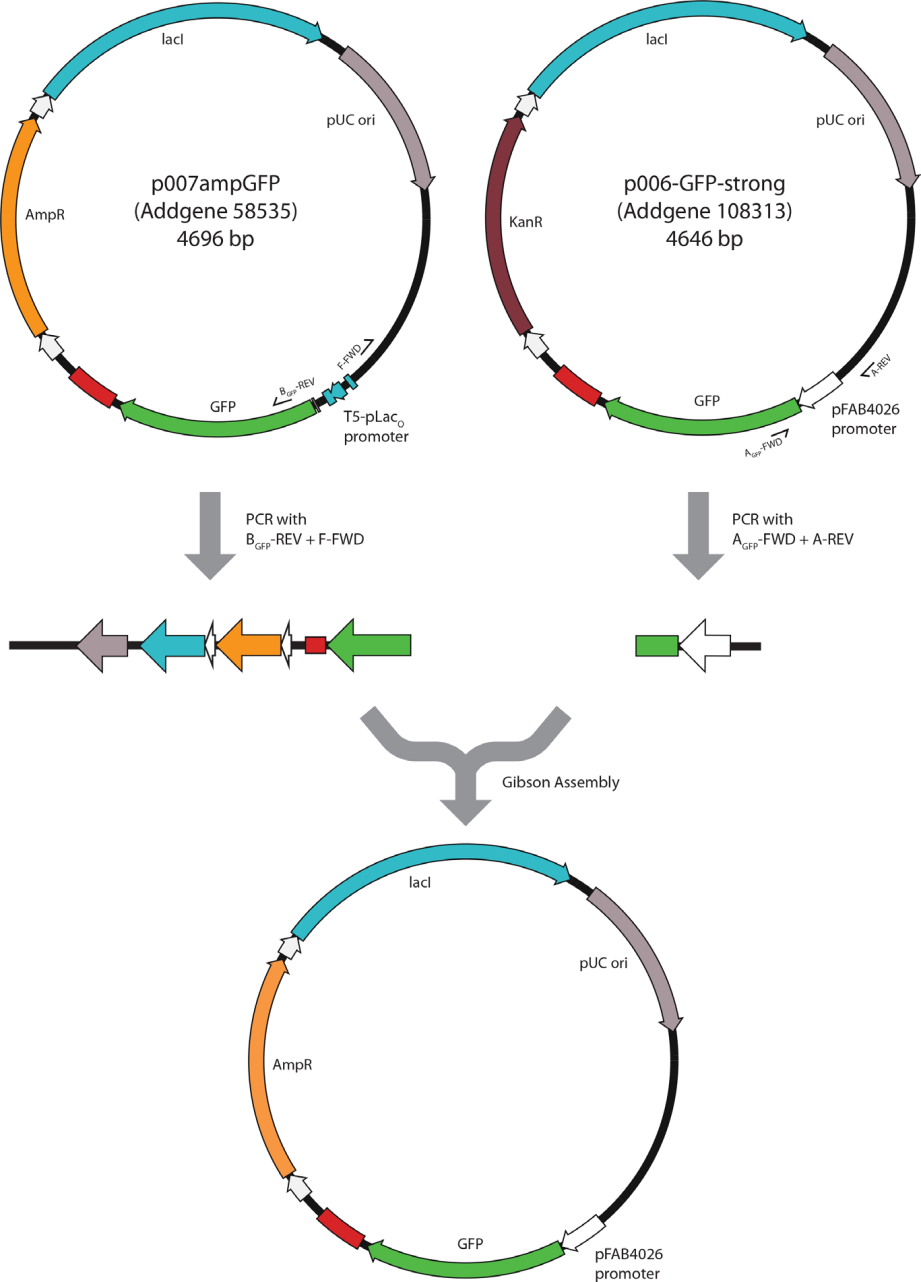
Example of novel plasmid assembly using existing unigems plasmids as sources of vector and insert parts with PCR and Gibson assembly. See text for details.

This principle can be illustrated with the following example (Fig. 2). Let our starting point be the p007ampGFP plasmid (Addgene 58535), where a reporter gene (GFP) is under control of the lactose-inducible promoter (T5-pLac_O_) and the plasmid contains an ampicilin-resistance gene. To replace the lactose-inducible promoter with a constitutive one (for example, a BIOFAB promoter pFAB4026, available in p006-GFP-strong (Addgene 108313)), we would run two PCR reactions: one to generate the recipient vector and one to generate a donor part (p005ampGFP and p006-GFP-strong, respectively).

We would use primers B_GFP_-REV and F-FWD to generate linearized recipient plasmid and primers A_GFP_-FWD and A-REV to generate the donor part. Because the primers B_GFP_-REV and A_GFP_-FWD, as well as F-FWD and A-REV are overlapping, both of their PCR products are directly usable for Gibson assembly. After purification and quantification, they can be mixed in appropriate molar proportions to assemble a new plasmid.

The same principle can be used to exchange or remove the entire reporter gene, antibiotic resistance gene, origin of replication or the repressor gene between the plasmids or with a newly generated or synthesized part. The combination of different promoters, reporters and antibiotic resistances ensures identification of a successful assembly. In our example, the colonies would be grown on ampicillin-containing media with no lactose or IPTG to identify GFP-fluorescent bacteria, indicating correctly assembled plasmids.

Because primers A_GFP_-FWD and B_GFP_-REV are located inside the ORFs of the reporter genes, they are specific to these genes. However, change of the promoter could also be achieved by inserting the entire ORF of the reporter gene combined with the promoter and part of the terminator. In this case, primers A-REV and B-FWD would be used and they would be identical for every reporter-promoter combination to be exchanged in any of the unigems plasmids.

Following assembly, we experimentally verified the characteristics of the plasmids.

### Constitutive promoters

**Fig. 3.**
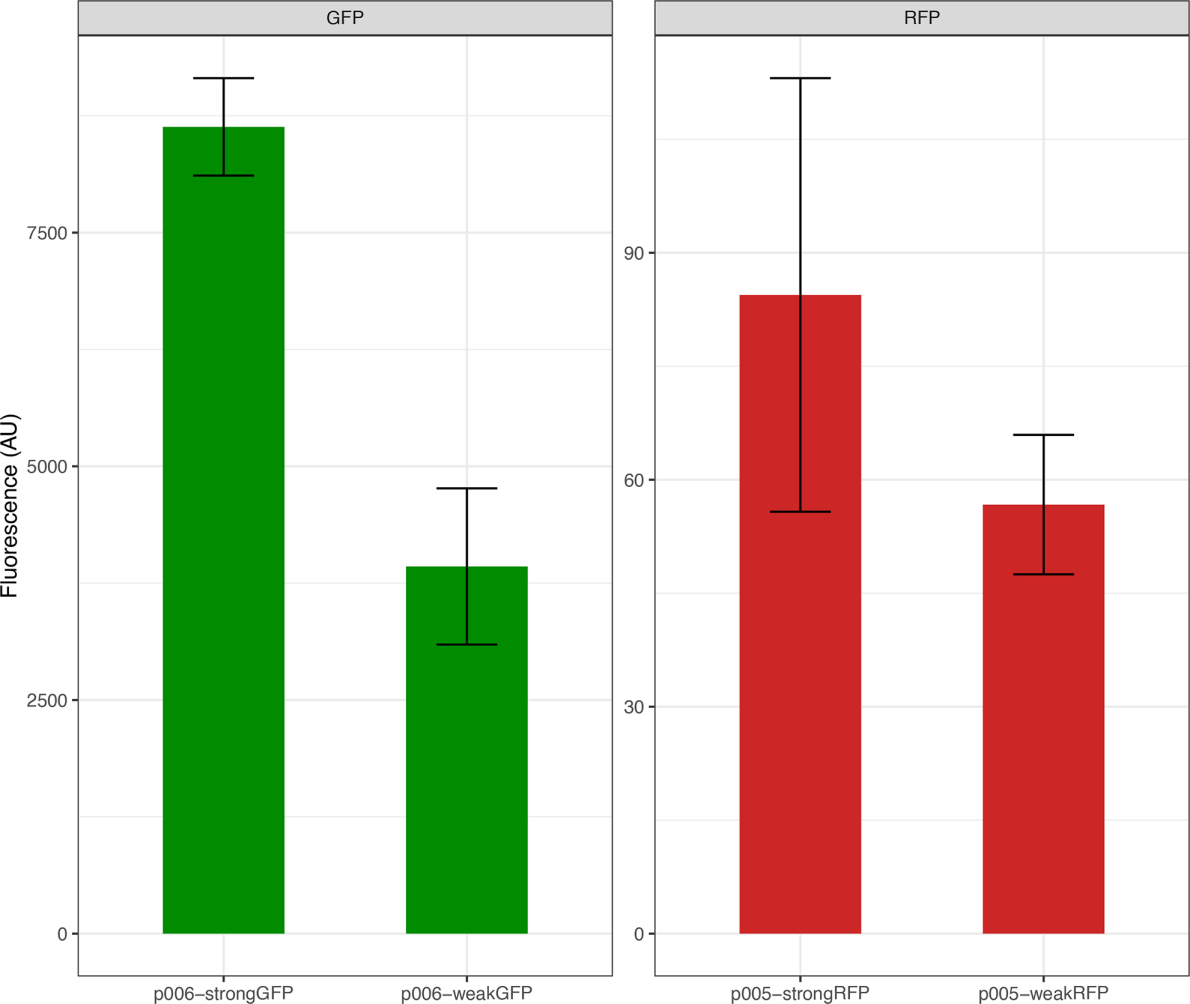
GFP fluorescence of three replicates of each p006-strongGFP and p006-weakGFP and RFP yellow fluorescence of p005-strongRFP and p005-weakRFP. Note the difference in scale in GFP vs RFP: the lack of differences between strong and weak RFP fluorescence is due to a mismatch between its optimal excitation wavelength (550 nm) and the 488 nm excitation laser in the flow cytometer (see Discussion).

GFP and RFP reporter genes, placed under the control of either a strong or a weak promoter (pFAB4026 and pFAB4282 for GFP and pFAB4005 and pFAB4024 for RFP, respectively) (BIOFAB collection [26]) were analysed using a flow cytometer on three replicate samples each (separate colonies from the same transformation event). To characterize the properties of the fluorescent proteins, we used a spectrofluorometer to test a range of excitation wavelengths for both GFP and RFP. We found that the GFP can be excited between 350 to 420 nm (with a peak at 395 nm) for emission at 506 nm. The RFP can be excited between 480 and 505 nm (with a peak at 505 nm) for emission at 560 nm. The fluorescence levels of the reporter genes were measured using the Guava easyCyte 5HT, with cells grown in liquid culture (Fig. 3).

### Inducible promoters and logic gate

**Fig. 4.**
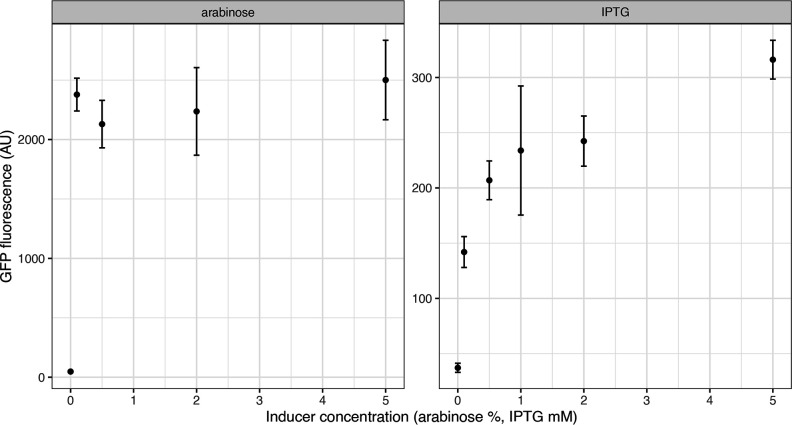
GFP fluorescence of three replicates of p006-strongGFP under arabinose-inducible p006-pBADGFP or IPTG-inducible p006kanGFP.

To demonstrate the inducibility of the pBAD and T5-pLacO promoters, we measured fluorescence of GFP with an increasing concentration of inducers, arabinose (0–5 %) and IPTG (0–5 mM). We observed clear activation depending on the inducer. pBAD exhibits a binary-like induction, where already at 0.1 % concentration of arabinose it produces 90 % of fluorescence observed at 5 % arabinose. In contrast, T5-pLacO produces a more dose-dependent pattern of induction (Fig. 4).

**Fig. 5.**
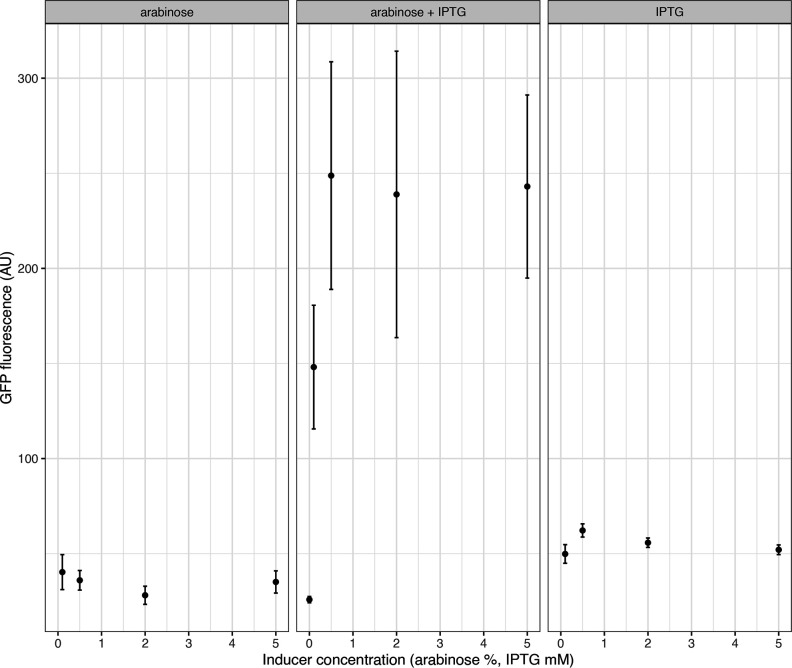
GFP fluorescence of three replicates of p006-GFP-Logic-AND induced by individual inducers (arabinose or IPTG) or by both inducers.

We also used combined lactose- and arabinose-inducible promoter based on design by Cox and colleagues [27]. This promoter acts as a biological AND gate, where both inputs (IPTG and arabinose) are necessary to activate the output – expression of the GFP. We tested the performance of the logic gate with arabinose only, IPTG only and both, at increasing concentrations. GFP fluorescence does not increase significantly in the presence of either IPTG or arabinose alone, but is clearly inducible in the presence of both inducers (Fig. 5).

### Olfactory construct

The p006-Banana-Late construct is identical to the *Eau de Smell* described by Dixon and colleagues [17] and produces ATFI enzyme (alcohol acetyltransferase I) that converts isoamyl alcohol to isoamyl acetate, which has a strong banana odour. ATFI production is controlled by the osmY stationary phase promoter, therefore the banana odour can only be detected once the cells reach the stationary growth phase. The construct was tested in broth culture.

## Discussion

We have assembled and characterized a set of plasmids that enable out-of-the-box investigations of gene expression control and straightforward extensibility with PCR and Gibson assembly. The phenotypes presented are clearly distinguishable and interpretable in an educational context rather than being designed and tested to produce precise quantitative output. For instance, Mutalik and colleagues reported (2013) that the relative difference in GFP fluorescence driven by BIOFAB promoters pFAB4026 and pFAB4282 is sevenfold vs threefold in our characterization (Fig. 3), the discrepancy that can reasonably be attributed to differences in bacterial chassis, fluorescent marker sequence and culture conditions. The minimal signal from RFP fluorescence shown on Fig. 3 is due to a mismatch between its optimal excitation wavelength (550 nm) and the 488 nm excitation laser in the flow cytometer. The GFP’s excitation and emission reported here are similar to those reported by Heim and Tsien [28] for an unmodified protein [28]. The RFP’s excitation and emission spectra behave similarly to that quantified by Baird *et al*. [29]. Overall, the performance of the plasmids in the K12 *E. coli* strain DH5alpha is suitable for demonstrating the quantitative differences between various promoters and inducers, including those in the bi-inducible promoter.

**Fig. 6.**
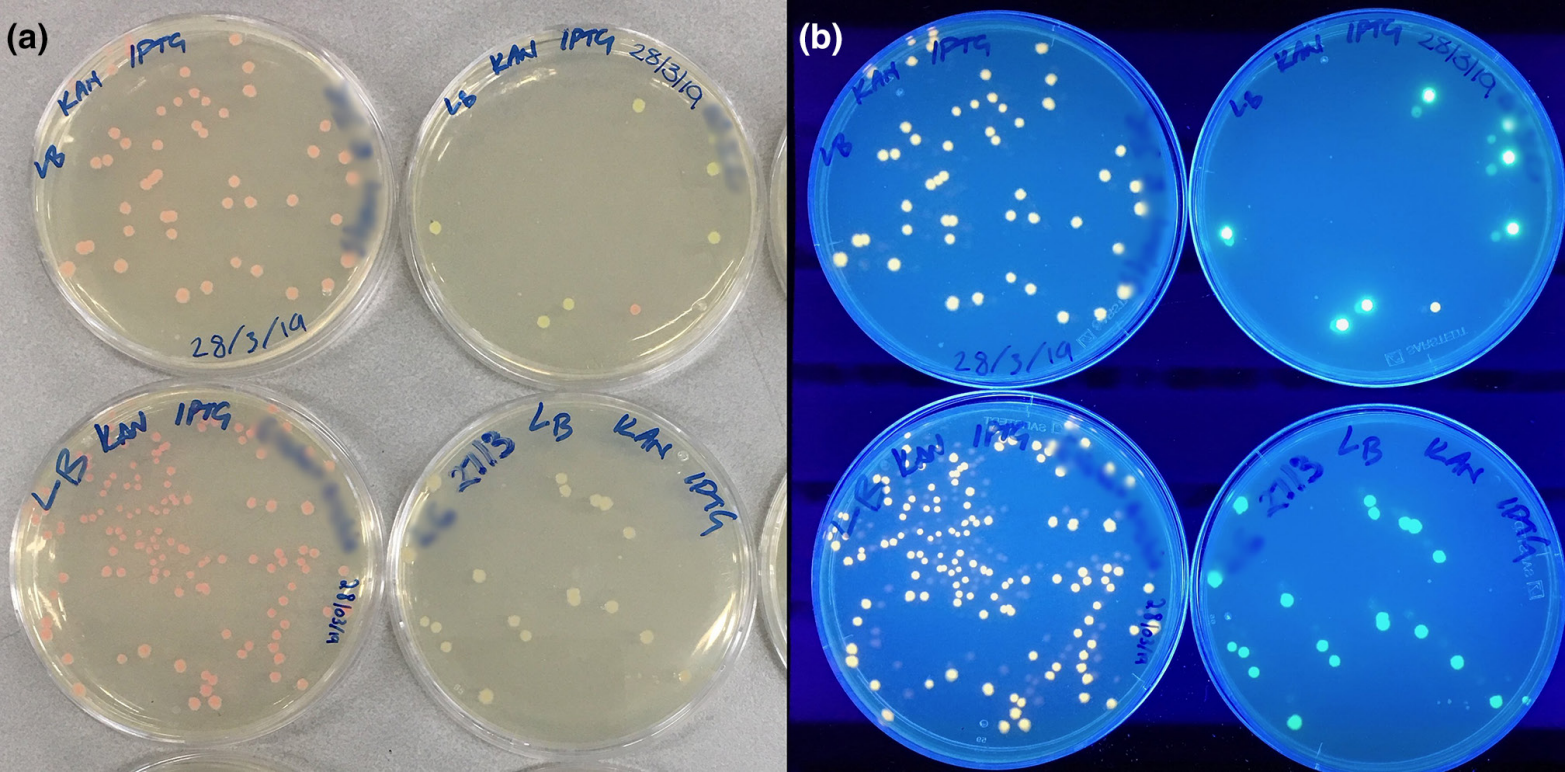
Images of p005-kan-RFP (left column) and p006-kan-GFP (right column) in daylight (**a**) and on a benchtop UV transilluminator (excitation wavelength 395 nm) (**b**). Transformed DH5α cells were grown with 50 µg ml^−1^ of kanamycin and induced with 100 mM IPTG. The single RFP-expressing colony on the top right plate is due to a mix of plasmids used by the students in this transformation. Photographs were taken with a mobile phone by JB.

A standard benchtop UV transilluminator or a keyring UV torch, with excitation at ~400 nm, is the only equipment required to verify the expression of the reporter genes. Expression of RFP is also visible in daylight without any equipment (Fig. 6). Fluorometer or flow cytometer are only needed to quantify the expression level of GFP or RFP.

We (AS, JB, JS, DM) have been using the unigems plasmids in a variety of contexts, ranging from outreach events, where participants transform *E. coli* with p006kanGFP or p005kanRFP using a 15 min protocol to observe a spectacular fluorescence after an overnight incubation to undergraduate research projects, where students have to design a new compatible part (e.g. a NOT gate, or a quorum-sensing system) and characterize its functions following a successful assembly. The plasmids themselves were first tested in two synthetic biology workshops run for prospective members of iGEM teams. Students’ feedback from this course is included in the report available in the Figshare repository (https://figshare.com/articles/media/Unigems_report_from_a_synthetic_biology_workshop_for_undergraduates/14627211). Since deposition in Addgene, Unigems plasmids have enjoyed a stable stream of requests.

The availability and use of GMOs is regulated in almost all jurisdictions [30–32]. In the UK, at the time of writing, the use of the unigems plasmids needs to comply with the UK’s Health and Safety Authority’s Genetically Modified Organisms (Contained Use) Regulations 2014 (https://www.hse.gov.uk/pubns/books/l29.htm). These guidelines allow for exemptions if the constructs and parts have a long history of safe use, but they are still restrictive compared to the US regulations, which only require that constructs do not pose an unreasonable risk (Toxic Substances Control Act, Environmental Protection Agency, https://www.epa.gov/laws-regulations/summary-toxic-substances-control-act). Therefore, use of the unigems system within high school classrooms would be possible in some jurisdictions, but it would pose an administrative burden in most. On the other hand, as most undergraduate biology teaching facilities already comply with GMO containment requirements, unigems can be straightforwardly employed at higher education institutions.
